# Genetic diversity of livestock-associated MRSA isolates obtained from piglets from farrowing until slaughter age on four farrow-to-finish farms

**DOI:** 10.1186/s13567-014-0089-4

**Published:** 2014-09-13

**Authors:** Marijke Verhegghe, Florence Crombé, Larissa J Pletinckx, Freddy Haesebrouck, Patrick Butaye, Lieve Herman, Marc Heyndrickx, Geertrui Rasschaert

**Affiliations:** Institute for Agricultural and Fisheries Research (ILVO), Technology and Food Science Unit, Food safety research group, Brusselsesteenweg 370, 9090 Melle, Belgium; Department of Pathology, Bacteriology and Avian Diseases, Ghent University, Faculty of Veterinary Medicine, Salisburylaan 133, 9820 Merelbeke, Belgium; Department HIVB, Catholic University College South-West-Flanders (KATHO), Wilgenstraat 32, 8800 Roeselare, Belgium; Department Biosystems, Division Gene Technology, Katholieke Universiteit Leuven, Kasteelpark Arenberg 30, 3001 Heverlee, Belgium; Department of Bacteriology and Immunology, Veterinary and Agrochemical Research Centre (VAR), Groeselenberg 99, 1180 Brussels, Belgium

**Keywords:** LA-MRSA, Molecular typing, Epidemiology, Pigs, Genetic diversity

## Abstract

During a previous longitudinal study, performed on four farrow-to-finish farms (A to D), samples were taken from twelve sows, their offspring, and the environment on various occasions over six months to study the MRSA presence. During the present study, a selection of the obtained MRSA isolates were typed by multiple-locus variable-number tandem-repeat analysis (MLVA), Pulsed Field Gel Electrophoresis (PFGE), *spa* typing, and SCC*mec* typing to study the genetic diversity of LA-MRSA isolates and to determine possible MRSA sources for pig(let)s. PFGE, *spa* typing, and SCC*mec* typing revealed the presence of one or few dominant genotype(s) per farm. In contrast, 212 MLVA types were detected on the four farms, forming one cluster on farm A, three on farm B, four on farm C and two on farm D. The genotype, found on farm A was unique for this farm. Farms B, C and D shared one cluster. In general, MLVA types from these clusters were isolated from piglets, sows, and the environment on various sampling events. Piglets carried MLVA types both related and unrelated to their mother sows’ MLVA types at farrowing and onwards. In conclusion, molecular typing revealed that within a farm one or a few dominant strain(s) are widespread. Potential MRSA sources for piglets were mother sows, the environment and other piglets.

**Electronic supplementary material:**

The online version of this article (doi:10.1186/s13567-014-0089-4) contains supplementary material, which is available to authorized users.

## Introduction

Since Voss et al. [1] first described a new methicillin-resistant *Staphylococcus aureus* (MRSA) type in a pig farmer and his pigs, this livestock-associated MRSA (LA-MRSA) has been isolated from different livestock animals (especially pigs) and from humans having close contact with them [2,3]. LA-MRSA rarely causes infections in pigs. Pigs are regarded as a potential source of MRSA for the human population, although at present the risk of transmission into the general human population appears low [4,5]. To prevent dissemination of LA-MRSA in animals and humans, the MRSA load on pig farms should be reduced or eliminated. Implementation of, for example, hygienic measures might be useful but before implementing such measures, the main MRSA types and sources on a farm should be identified.

Molecular typing is very useful to investigate sources and vectors of pathogens. At present, different typing methods are available to study the spread of MRSA strains. Methods, such as multilocus sequence typing (MLST) and *spa* typing indicated that LA-MRSA, which is mostly MRSA ST398, is a rather clonal type with a limited set of *spa* types [6]. SCC*mec* cassette types IVa and V are mainly identified in these isolates. MRSA ST398 appeared non-typeable when using the gold standard Pulsed Field Gel Electrophoresis (PFGE) protocol with *Sma*I restriction which is due to a methylation of its restriction site [7]. At present other restriction enzymes such as *BstZ*I, *Apa*I, and *Cfr*9I have been used instead [8,9]. A more recent method to discriminate between clonal isolates is multiple-locus variable-number tandem-repeat analysis (MLVA). This method has been used for epidemiological studies of human *S. aureus* isolates or *Salmonella* isolates. MLVA is more discriminatory than MLST and *spa* typing and detects short-term evolution within strains [8]. This could be a good method in a pig farm setting where other typing techniques show too little variation between isolates.

In the present study, MLVA typing was used for the first time on a large subset of LA-MRSA isolates, obtained from a previous longitudinal study on four farrow-to-finish farms [10]. Besides MLVA, the more classical typing methods (*spa* typing, SCC*mec* typing, and PFGE) were used as well to confirm the MLVA typing results. The main goal of the present study was to investigate the genetic diversity of LA-MRSA isolates from sows, their piglets and their environment from farrowing till slaughter age by mainly using MLVA typing to gain insight into this diversity on pig farms and to identify potential MRSA sources on the basis of genetic relationships.

## Material and methods

### Isolate collection

From July 2009 to December 2010, four farrow-to-finish farms (A to D) were sampled during a six-month period [10]. As sampled animals were not harmed, and according to the European Convention for the Protection of Vertebrate Animals used for Experimental and Other Scientific Purposes ETS 123, no animal utilization protocol was needed [11]. In short, on each farm, nasal swabs were collected from 12 sows and their offspring. From farrowing until weaning, the sows were sampled in the nursing unit on six occasions on farms A, B and C and on three occasions on farm D. Sampling of the piglets occurred from farrowing until slaughter age on 10 (farms A and B) and 11 (farms C and D) time points. On each sampling day environmental samples were also taken from the wall, floor, and air of one pen per stable. Two sites were present on farm C: piglets were born on site 1 where they resided until they were approximately five weeks old after which they were transported to the second site where they stayed until slaughter age. On site 1, the animals received a promycine and amoxicillin treatment in the growing unit, which was repeated upon arrival on site 2. Verhegghe et al. reported on two trends after bacteriological analysis of the obtained samples [10]. Farms A and B were defined as low colonization farms whereas farms C and D as high colonization farms. On the low colonization farms, MRSA was isolated sporadically from the sows and piglets in the nursing unit. The colonization percentage of the piglets increased at the end of the stay in the growing unit and remained high till slaughter age. None of the animals on these farms was a persistent MRSA carrier. Intermittent MRSA carriage was observed in 99% and 100% of the pigs of farm A and B, respectively. Thirty-three percent of the farm A sows and 17% of the farm B sows also were an intermittent MRSA carrier. On the high colonization farms, the colonization percentage of the sows and piglets in the nursing unit was high and remained high throughout the sampling events. No non-MRSA carriers were found on these farms. On farm C, 25% of the sows and 47% of their offspring were persistent MRSA carriers, whereas on farm D 92% of the sows and 37% of their offspring showed a persistent carriage. The remaining animals were intermittent carriers. In total, 3450 isolates (one isolate per positive sample) were collected on the four farms: 262 and 407 isolates on farms A and B, respectively, and 1284 and 1497 isolates on farms C and D, respectively. To confirm the presence of livestock-associated MRSA, the CC398 specific PCR as described by Stegger et al. was performed on a selection of isolates [12]. This selection was equal to the isolates chosen for Pulsed Field Gel Electrophoresis (PFGE) (see further).

Due to the large number of obtained isolates, a selection was made and a total of 960 isolates were genetically characterized. From each farm, all sow isolates (A: *n* = 4, B: *n* = 4, C: *n* = 45 and D: *n* = 22) were included. To obtain a representative collection of piglet isolates, the selection of these isolates was different for the low and high colonization farms. On the low colonization farms (A and B), few MRSA was isolated from the piglets throughout the six-month period. Few sows carried MRSA in the nursing unit of both farms. From each sow a variable number of piglets was chosen, being piglets with the highest MRSA isolation rate in the litter. From these, piglets with remarkable colonization profiles were selected to gain insight into the genetic diversity of MRSA during the life of one piglet (e.g. piglets carrying MRSA at one sampling event in the nursing unit, being MRSA-negative the following sampling event(s) and becoming an MRSA carrier later during their life, for example, in the growing or finishing unit). In addition, comparisons within and between litters could be carried out. On farm A, two to six piglets per sow were chosen whereas on farm B, three to six piglets per sow. In total, 44 piglets of farm A and 45 piglets of farms B were chosen, resulting in 127 and 143 isolates, respectively. On the high colonization farms (C and D), MRSA was isolated from most piglets on all sampling events. Again, a selection of piglet isolates was made. Eight and nine out of twelve sows of farms C and D, respectively, were chosen. From these sows, all isolates from three to four piglets per sow were selected for the MLVA typing (29 piglets representing 278 isolates on farm C and 30 piglets representing 276 isolates on farm D). Besides gaining more insights into genetic diversity during a piglet’s lifetime and to observe the genetic diversity within one litter and between litters, this selection made it possible to compare the genetic diversity of isolates between the low and high colonization farms. To determine whether the environment might act as a MRSA source, from the environmental isolates, all wall isolates were also included (A: *n* = 4, B: *n* = 8, C: *n* = 27 and D: *n* = 22).

### Molecular typing

On all 960 isolates MLVA was performed on the repeat regions of five genes being *clf*A, *clf*B, *sdr*C, *sdr*E, and SIRU21. A modified protocol of the method of Rasschaert et al. [8] was used: fluorescent primers were used to allow capillary electrophoresis. Fragment sizing of the PCR products was done on a 3130xl Genetic Analyzer (Applied Biosystems/Hitachi, Hitachinaka-shi, Japan) using the Genescan™ 1200LIZ® size standard (4379950, Applied Biosystems, Warrington, UK). The obtained patterns were transformed into numeric codes using the MLVA plugin in Bionumerics (Bionumerics version 6.5; Applied Maths, St.-Martens-Latem, Belgium). Categorical analysis using the unweighted pair cluster method using averages (UPGMA) was performed. A tolerance of 0% was used in contrast to the described tolerance of 1% [8] to obtain the MLVA types per farm. This adapted protocol needed validation. In each run the same MRSA ST398 strain (MV-162) was added as positive control and we observed a good inter and intra-run repeatability (data not shown). For convenience and clear representation of the results each MLVA numeric code (a string of five integers) was converted to a MLVA type with a unique number (for example: MLVA numeric code 32-46-38-38-2 of farm A was converted to MLVA type 11). In addition, a Minimum Spanning Tree (MST), based on the numeric code, was generated for all farms together (Additional file 1) and for each farm separately in Bionumerics. Due to the large variety of MLVA types per farm, clustering of these types was performed. A single and double locus approach was evaluated for clustering the MLVA types. The first approach clustered the dominant MLVA type with single locus variants (=MLVA types with one difference in one repeat region) and the double locus approach clustered the dominant MLVA type with MLVA types with two differences in the repeat regions. The single locus approach was selected (data not shown). Each cluster consisted of a dominant MLVA type with closely related types, being single locus variants. For example, the predominant MLVA type 11 (32-46-38-38-2) of farm A was closely related to eight other MLVA types, belonging to cluster A. The clusters were indicated on the MST of each farm. MLVA types containing only one isolate were defined as singletons.

Pulsed Field Gel Electrophoresis (PFGE) with *BstZ*I restriction (Promega, Madison, WI, USA), as described by Rasschaert et al. [8], was performed on 226 isolates in total: 41 of farm A (4 sow, 34 piglet, and 3 environmental isolates), 43 of farm B (4 sow, 31 piglet, and 8 environmental isolates), 86 of farm C (16 sow, 59 piglet, and 11 environmental isolates) and 56 of farm D (8 sow and 48 piglet isolates). The isolates were chosen to represent as many of the dominant MLVA cluster(s) and as many other non-dominant MLVA types per farm as possible. The sow isolates belonged to different MLVA types. From each of these sows, one or two piglets were chosen with as many isolates (of various time points) as possible. In addition, the latter isolates belonged to as many MLVA types as possible. The obtained restriction profiles were analyzed in Bionumerics version 6.5 using UPGMA with the Dice coefficient (tolerance 1%, tolerance change 1%, and optimization 1%). Pulsotypes were determined based on a delineation level of 97%, which corresponds to the difference in the presence or absence of at least one band as described by Rasschaert et al. [8]. The pulsotypes were given a roman number. When a pulsotype was detected on more than one farm, the same roman number was used.

*Spa* typing and SCC*mec* typing was performed on 11, 11, 25, and 24 isolates of farms A through D, respectively. These isolates were chosen from each obtained pulsotype and each sampling event or farm unit was represented as much as possible. The Ridom StaphType standard procedure [13] was used for *spa* typing and the *spa* type was determined using the Ridom StaphType software (Ridom GmbH, Würzburg, Germany). Three different protocols were used for SCC*mec* typing [14–16] and results were combined to obtain the SCC*mec* type. When a non-typeable SCC*mec* type was found, the method of Kondo et al. [17] was used and the *mec* and *ccr* complex is given.

## Results

### Genetic diversity on the four farms

The CC398-specific PCR indicated that the all typed isolates belonged to CC398, the animal-associated clonal complex.

**Figure 1:**
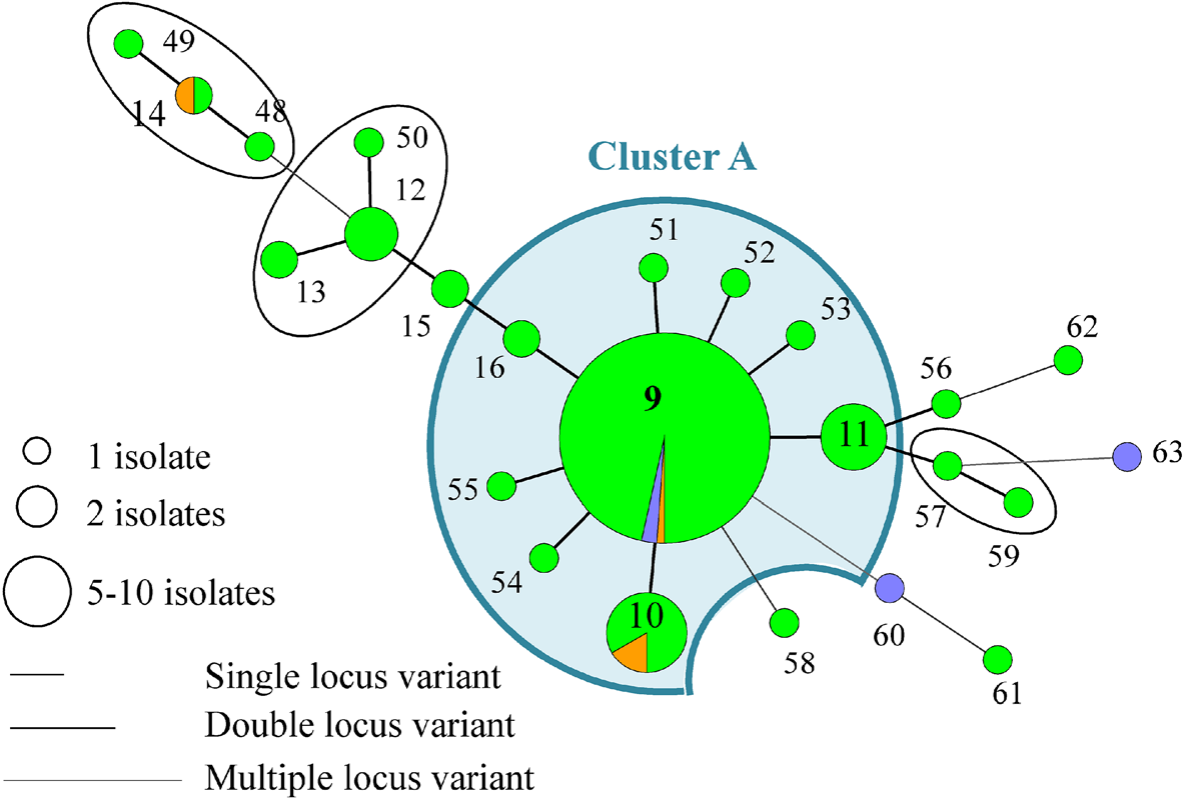
**Minimum Spanning Tree of the farm A MLVA types.** The MLVA types are indicated by numbers, according to origin (green: piglet, orange: sow and blue: wall). The dominant cluster A of the farm is indicated in a coloured sphere, whereas the non-dominant clusters are indicated in black spheres.

In total, 212 MLVA types were detected in the 964 isolates, originating from the different farms. Each MLVA type consisted of a five-string numeric code and received an unique number (Additional files 1 and 2). Closely related MLVA types (single locus variants = MLVA types differing in one repeat region) were clustered. For example, the predominant MLVA type 11 (32-46-38-38-2) of farm A was closely related to eight other MLVA types, belonging to cluster A (Figure 1, Table 1).

**Table 1:** **Summary of the MLVA results**

		**Farm**							
		**Farm A**		**Farm B**		**Farm C**		**Farm D**	
**MLVA cluster**	**Dominant MLVA type(s)** ^**a**^	**# MLVA types in cluster**	**# isolates in cluster/total**	**# MLVA types in cluster**	**# isolates in cluster/total**	**# MLVA types in cluster**	**# isolates in cluster/total**	**# MLVA types in cluster**	**# isolates in cluster/total**
A	32-46-38-38-2	9	113/135	-	-	-	-	-	-
B	33-57-37-7-3	-^c^	-	16	89/155	5	66/355	12	157/326
	33-57-37-6-3								
C	34-57-34-7-3	-	-	-	-	15	105/355	-	-
D	34-59-38-8-4	-	-	-	-	4	57/355	6	131/326
E	35-59-35-8-4	-	-	-	-	4	54/355	-	-
F	32-55-35-5-3	-	-	4	12/155	-	-	-	-
G	33-57-36-7-3	-	-	2	11/155	-	-	-	-
Other clusters^b^		8	14/135	11	11/155	18	20/355	9	12/326
Non-clustered		7	8/135	24	32/155	48	53/355	22	26/326

^a^VNTR code of the repeat region of the 5 genes *clf*A, *clf*B, *sdr*C, *sdr*E, and SIRU21.

^b^There are 3, 5, 9, and 4 other clusters on farm A to D, respectively.

^c^-: not detected on the farm.

For each of the seven dominant clusters (=related MLVA types grouped together), the dominant MLVA type(s) is/are given. Per farm, the dominant cluster(s) is indicated. MLVA clusters containing less than 10 isolates were grouped as “other clusters” (3, 5, 9, and 4 clusters on farm A to D, respectively). Last, the number of isolates belonging to a non-clustered MLVA type is given. In addition, the number of MLVA types belonging to each cluster and the number of isolates per cluster are shown per farm.

**Figure 2:**
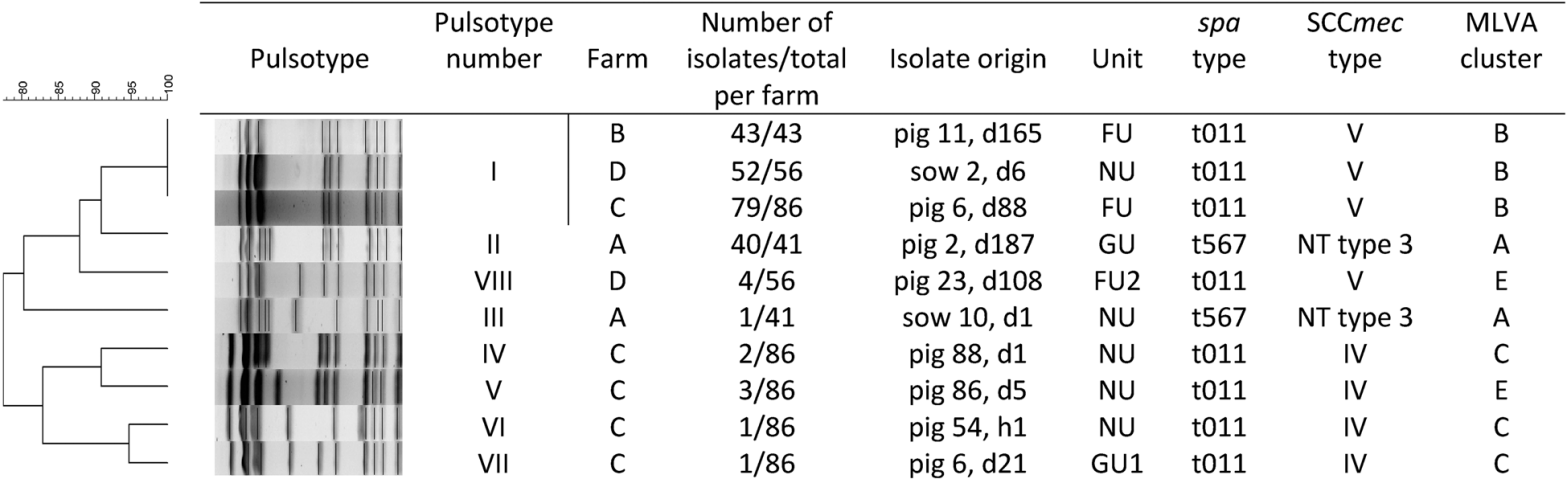
**Dendrogram containing the pulsotypes obtained on the four farms.** Per pulsotype, one isolate is shown as an example. Consecutive the dendrogram, pulsotype pattern, farm and number of isolates belonging to the pulsotype on the total number of typed isolates per farm are shown. For each example, the isolate origin, unit (NU: nursing unit, GU: growing unit, GU1: growing unit 1, FU: finishing unit, FU2: finishing unit 2), *spa* type, SCC*mec* type (NT *type* 3: *mec*A complex NT/*ccr* complec C) and MLVA cluster are given.

**Figure 3:**
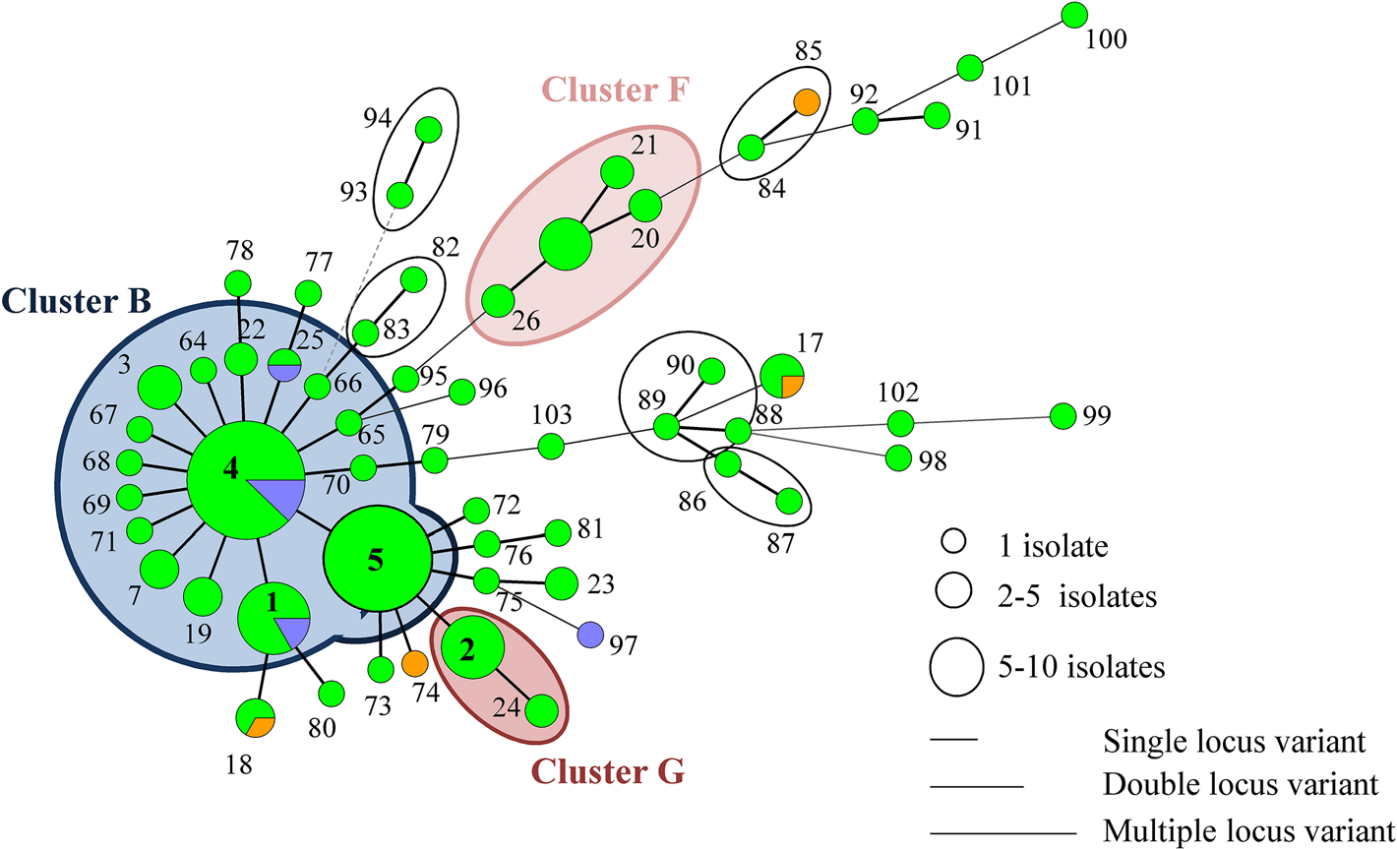
**Minimum spanning tree of the farm B MLVA types.** The MLVA types are indicated by numbers, according to origin (green: piglet, orange: sow and blue: wall). The dominant clusters B, G, and H of the farm are indicated in coloured spheres, whereas the non-dominant clusters are indicated in black spheres.

**Figure 4:**
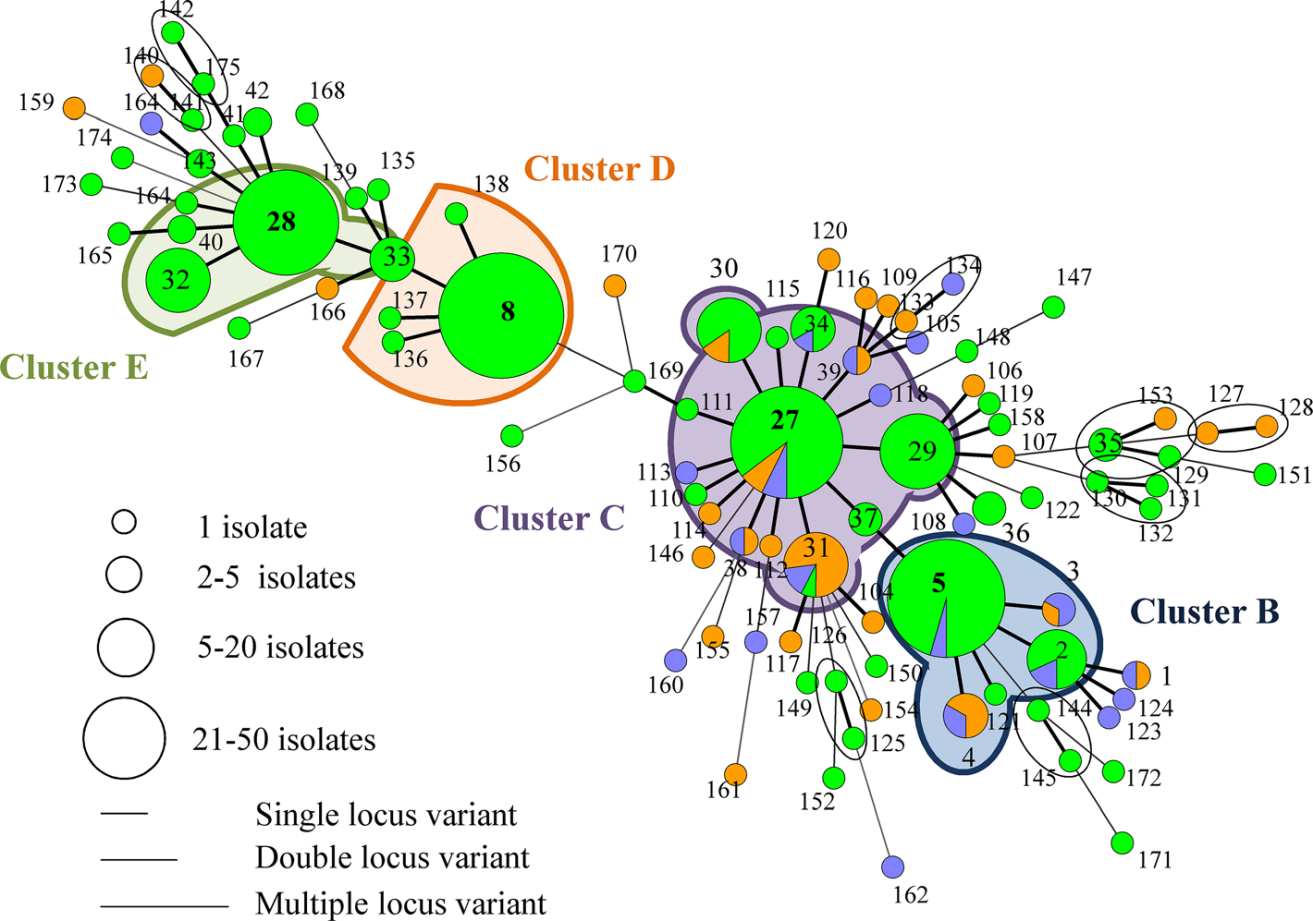
**Minimum spanning tree of the farm C MLVA types.** The MLVA types are indicated by numbers, according to origin (green: piglet, orange: sow and blue: wall). The dominant clusters B, C, D, and E of the farm are indicated in coloured spheres, whereas the non-dominant clusters are indicated in black spheres.

**Figure 5:**
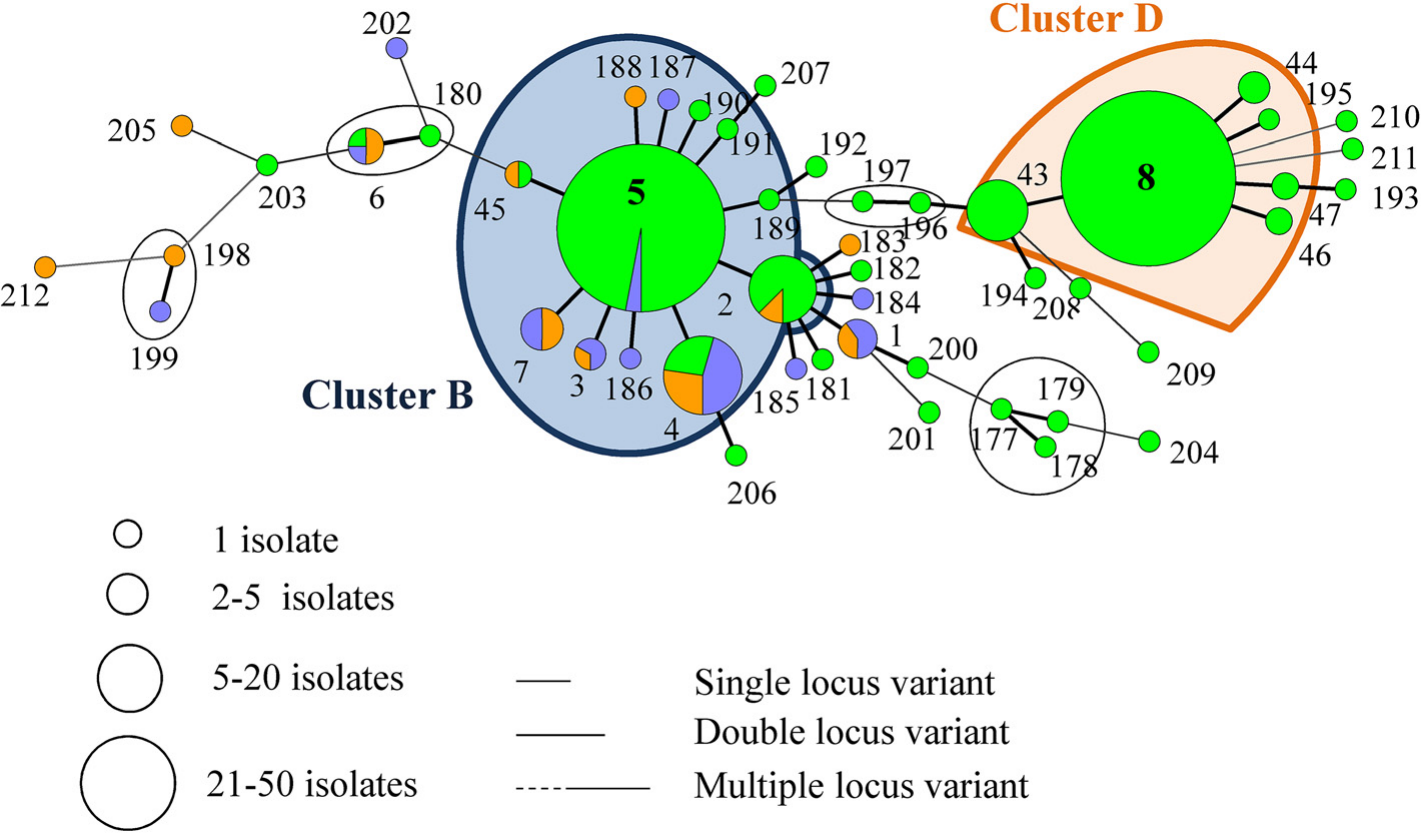
**Minimum spanning tree of the farm D MLVA types.** The MLVA types are indicated in numbers, according to origin (green: piglet, orange: sow and blue: wall). The dominant clusters B and D of the farm are indicated in coloured spheres, whereas the non-dominant clusters are indicated in black spheres.

On farm A, 24 MLVA types were detected of which 9 clustered in one predominant cluster A (Table 1, Figure 1). *Spa* type t567 was detected on the different pigs throughout the sampling events at the farm in combination with a non-typeable (NT) SCC*mec* cassette type (*mec*A complex NT/*ccr* complex C). In addition, one predominant pulsotype was found on this farm (II, 98% of the tested isolates) (Figure 2; Additional file 3). Fifty-seven MLVA types were observed on farm B, of which 16 were clustered in the predominant cluster B. The two other dominant clusters (F and G) each contained four and two MLVA types, respectively (Table 1 and Figure 3). One pulsotype, *spa* type t011, and SCC*mec* type V was present in the isolates of this farm belonging to various animals on various sampling events (Figure 2; Additional file 3). The tested isolates of farm C belonged to 94 MLVA types (Figure 4). Four predominant MLVA clusters (B, C, D, and E) were present, each consisting of 5, 15, 4, and 3 MLVA types, respectively (Table 1). *Spa* type t011 was detected and the isolates (originating from four animals on various sampling events) carried SCC*mec* type IV (45%) or V (55%). Both SCC*mec* cassette types, present on farm C, were found in the isolates of one animal, as shown in the example in Table 2. SCC*mec* type IV was present in MLVA clusters C and E whereas type V was present in MLVA clusters B and D (Table 2). Five pulsotypes (predominant type I in 92% of the tested isolates) were present on farm C (Figure 2; Additional file 3). Both SCC*mec* cassette types were found in the predominant pulsotype I (Table 2). On farm D, 49 MLVA types, of which 12 and 6 were clustered in MLVA clusters B and D, respectively, were present (Table 1 and Figure 5). *Spa* type t011 and SCC*mec* type V were observed together with two pulsotypes (predominant type I in 93% of the tested isolates) (Figure 2).

**Table 2:** **Example of the molecular typing results of all isolates obtained from two sows (sows 1 and 9) and two piglets (pig 6 originating from sow 1 and pig 88 originating from sow 9) from farm C**

**Origin**	**Sampling event(s) (days after farrowing)**	**SCC** ***mec*** **cassette type**	**MLVA numeric code** ^**a**^	**MLVA cluster or type**	**Pulsotype**
Sow 1	d1	IV	33-55-35-1-3	127	I
	d3	V	33-57-37-6-3	B	I
	d5	IV	33-53-34-8-4	171	I
	d17	V	33-57-36-6-3	1	I
Pig 6	<d1, d1, d54, d88	V	33-57-37-7-3	B	I
	d3	V	33-57-36-7-3	B	I
	d7	V	31-53-35-6-3	152	I
	d5, d17, d33	V	34-59-38-8-4	D	I
	d21	IV	34-57-34-7-3	C	VII
Sow 9	<d1	IV	32-53-34-4-3	155	V
Pig 88	<d1	IV	35-48-35-8-4	E	I
	d1	IV	34-47-34-7-3	C	IV
	d3, d5, d17	IV	34-47-34-7-3	C	I
	d7	IV	34-47-33-7-3	36	I
	d21	IV	35-59-35-8-4	E	I
	d33	V	33-57-37-7-3	B	I
	d54, d88	V	34-59-38-8-4	D	I
	d172	V	26-59-38-8-4	139	I

^a^VNTR code of the repeat region of the 5 genes *clf*A, *clf*B, *sdr*C, *sdr*E, and SIRU21.

All isolates belonged to *spa* type t011. The origin, sampling event, SCC*mec* cassette type, the MLVA numeric code, MLVA cluster or MLVA type number in case of a non-clustered MLVA type, and pulsotype is given for all isolates of each animal. Isolates (originating from the same animal at different sampling events) with the same characteristics were grouped together).

MLVA cluster A, the remaining MLVA types, the two pulsotypes, *spa* type and SCC*mec* type were unique to farm A (Table 1, Figure 2, Additional file 1). Farms B, C, and D shared one MLVA cluster (cluster B, dominant MLVA types: 33-57-37-7-3 and 33-57-37-6-3) and pulsotype I (Table 1, Figure 2). Moreover, MLVA cluster D (dominant MLVA type: 34-59-38-8-4) was similar on both farms C and D (Table 1). In general, PFGE and MLVA typing were more discriminatory than *spa* typing (one *spa* type versus few pulsotypes and few MLVA clusters). When observing the PFGE results of MLVA cluster A, one sow isolate (sow 10, day 1) belonged to another pulsotype, which showed 87% similarity to the predominant pulsotype (data not shown). On farms C and D, the predominant pulsotype was detected in all MLVA clusters present at the farm and various other MLVA types. Isolates from MLVA clusters C and E were categorized in the remaining four pulsotypes (less than 95% similar to the dominant type), detected on farm C, whereas MLVA clusters B and D in the other pulsotype (90% similarity with dominant pulsotype) on farm D (Additional file 3).

### MLVA diversity on the low colonization farms (farms A and B)

**Figure 6:**
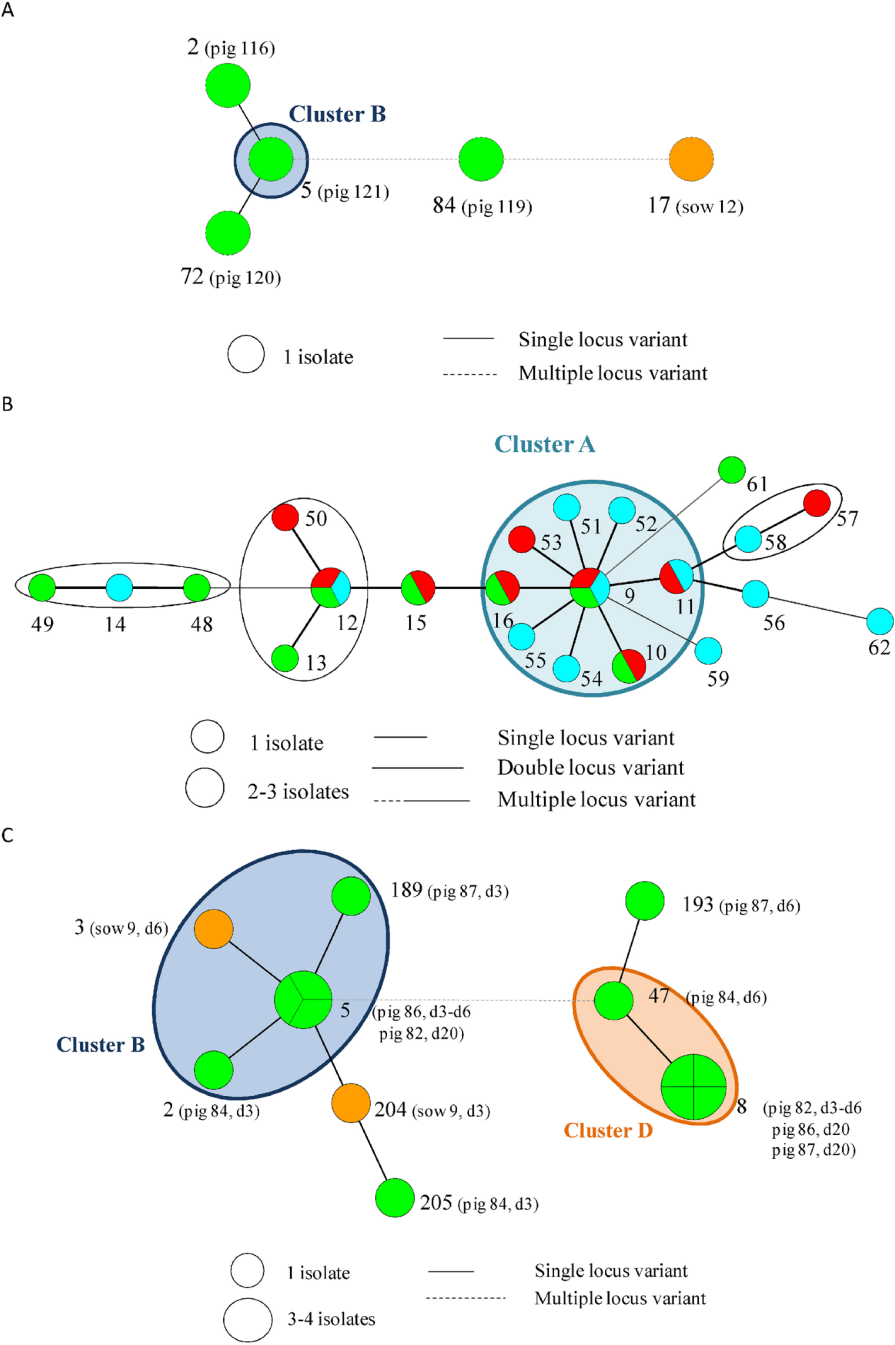
**Overview of some observations. A)** Piglets carrying non-related MLVA types to their mother sow (sow 12 and her offspring sampled one day after farrowing on farm B); **B)** Overview of the MLVA types found in the three units of farm A (green: nursing unit, red: growing unit and blue: finishing unit); **C)** Piglets carrying related MLVA types to their mother sow (sow 9 and her offspring sampled 3, 6, and 20 days after farrowing on farm D). In addition, piglets of the same litter carried various related and unrelated MLVA types.

The predominant MLVA cluster of both farms (cluster A and B on farms A and B, respectively) was found in isolates, originating from all units. On farm A and B, four and two sows, respectively, were MRSA-positive. On farm A three sows were colonized with MRSA belonging to MLVA cluster A (Figure 1). The two positive sows of farm B (sows 4 and 12) were colonized with MRSA of non-related MLVA types (Figure 3). Their offspring were not necessarily colonized with the same or closely related MLVA type as the one from the sow. For example, sow 12 (farm B) carried MLVA type 55 on the second sampling occasion. None of her offspring was colonized with this type or a closely related MLVA type (Figure 6A). After weaning, the piglets of farm A originating from different litters, were mingled upon entry in the growing unit (one large pen), whereas on farm B the litters remained together. Comparison of the MLVA types at the beginning and end of the growing period revealed the spread of some MLVA types throughout the cluster on farm A (Figure 6B). Novel MLVA types were also detected in the growing unit of which the majority belonged to the dominant MLVA cluster A (Figure 6B). The same observation (spread of MLVA types and the isolation of novel MLVA types) was made in the finishing unit of both farms (Figure 6B). Overviews of the MLVA typing results for each individual animal of farm A and B are shown in Additional files 4 and 5, respectively.

**Figure 7:**
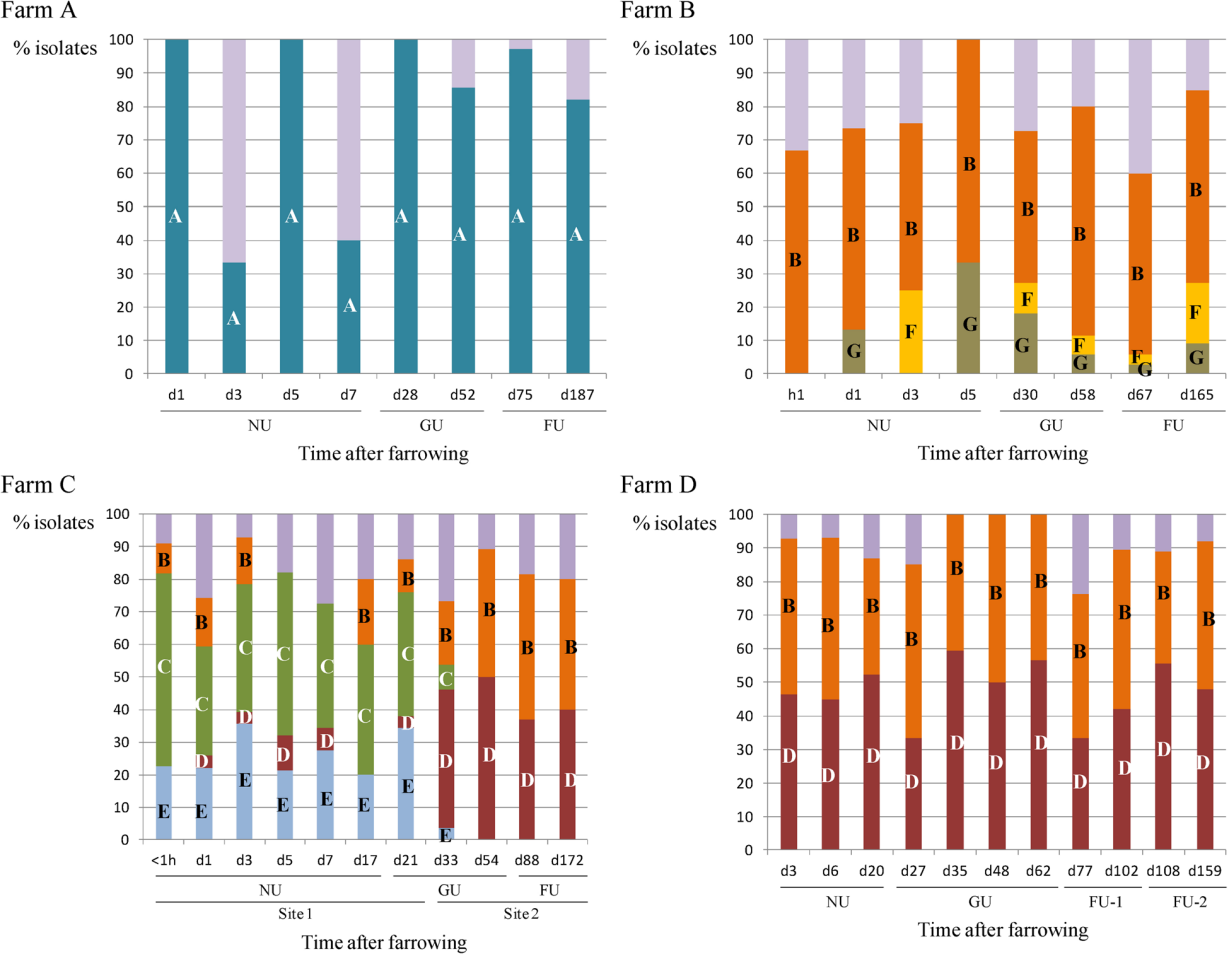
**Distribution of the MLVA clusters, observed on the four farms, at each time point after farrowing.** The cluster designation is shown in the bar. The non-dominant clusters and non-clustered MLVA types are given in the purple bar without indication. On farm **A**, no isolates were obtained within the hour and 17 days after farrowing, whereas on farm **B** 23 days after farrowing. The piglets of farm **C** were transported to the second site after a short stay in the growing unit of site 1. The animals received a promycin and amoxicillin treatment in both growing units. The pigs of farm **D** were moved from one finishing unit to another after a few weeks (h: hour, d: days, NU: nursing unit, GU: growing unit, FU: finishing unit).

From birth to slaughter age, most animals carried MRSA belonging to various MLVA types, which in general belonged to the dominant cluster of the farm. The distribution of the dominant MLVA clusters throughout the sampling events is shown in Figure 7. More than 80% of the farm A isolates belonged to the dominant MLVA cluster, except on two sampling events (three and seven days after farrowing) where < 40% of the animals carried an MLVA type belonging to this cluster (Figure 7). On farm B, the dominant MLVA cluster (B) was found evenly in the isolates throughout the sampling events. Clusters F and G were alternately detected in the isolates obtained from the nursing unit (Figure 7). From the four sows and one piglet of farm A only one isolate was obtained over time. Except for one piglet, all piglets carried MRSA isolates that belonged to the dominant cluster at least once during their lifetime. The MRSA isolates of 26 piglets were exclusively part of the dominant cluster A and 13 of these piglets carried the same MLVA type over time. The two sows of farm B carried MRSA categorized in different MLVA types. From one piglet of farm B only one isolate was obtained. The remaining 44 piglets all carried MRSA, belonging to the dominant cluster B at least once throughout their life. Thirty-six out of 44 piglets carried MRSA isolates belonging to different MLVA clusters and MLVA types over time.

Two out of four environmental isolates of farm A belonged to cluster A (Figure 1). On farm B, except for one, all environmental (wall) isolates belonged to cluster B (Figure 3).

### MLVA diversity on the high colonization farms (farms C and D)

On the high colonization farms, piglets and their mother sows often carried MRSA belonging to the same MLVA cluster. For example, sow 9 (farm D) was colonized with MRSA belonging to MLVA cluster B. All four piglets were colonized with cluster B MLVA types at least once in the nursing unit. Each piglet also carried MLVA types belonging to cluster E or unrelated types (Figure 6C). MRSA of piglets of the same litter did not always belong to the same MLVA type or MLVA cluster at different sampling events. As seen on farms A and B, most animals were colonized with MRSA of several (closely related and unrelated) MLVA types throughout their life. For each animal of farm C and D an overview of the individual MLVA typing results is shown in Additional files 6 and 7, respectively.

On farms C and D, none of the animals carried MRSA isolates belonging to the same MLVA type on all sampling events. From two and six sows of farms C and D, respectively, only one isolate was obtained. The MRSA isolates of one sow of farm C did not belong to any cluster. Two sows carried MRSA belonging to the same cluster during the sampling events. The MRSA isolates of five and two sows were retrieved from two and three clusters, respectively, over time. All MRSA isolates of one piglet of farm C belonged to two clusters. During the lifetime of four and seventeen piglets, the MRSA isolates were located in three and four clusters, respectively, over time. The isolates of seven piglets originated from more than four clusters during their life. One sow of farm D carried MRSA isolates originating from one cluster, two sows from two clusters and three sows from more than two clusters throughout the sampling events. Half of the piglets of farm D carried MRSA isolates belonging to clusters B and D during their life. From the other half of the piglets, MRSA was isolated that belonged to MLVA types not closely related to the dominant clusters. In general, the piglets carried MRSA isolates belonging to the four dominant clusters throughout the sampling events.

After a short stay in the growing unit, the pigs of farm C were transported to a second site where they resided until slaughter age. When observing the four dominant MLVA clusters of farm C a shift in these clusters was observed when the pigs were transported to the second site: on site 1 clusters C and E were more predominantly present than clusters B and D, whereas on site 2 clusters B and D were almost exclusively present in the pigs (Figure 7). On farm D, the two dominant MLVA clusters were equally found throughout the sampling events on the farm (Figure 7).

In most cases, the environmental isolates of farm C belonged to clusters B and C or these isolates belonged to singletons (MLVA types with only one isolate) (Figure 4). More than half of the environmental isolates of farm D were situated in cluster B. Five MLVA types (1, 3, 4, 6, and 7) were similar to the sows and environment of this farm (Figure 5).

## Discussion

At present, many molecular typing methods are available for the characterization of MRSA ST398. When methods such as MLST, PFGE, and *spa* typing are used, MRSA ST398 appears rather clonal. Seen this clonal nature, the use of other more discriminatory methods should be considered [3]. A more recently optimized method for MRSA ST398 typing is MLVA [8]. To our knowledge, this is one of the first studies where MLVA was used on a large collection of MRSA ST398 isolates from pigs.

Upon comparison of the typing methods used during the present study an important difference was observed between the results. After *spa* typing, SCC*mec* typing, and PFGE, we observed a small diversity of genotype(s) per farm (in general only one genotype). In contrast, a large and unexpected variety in MLVA types was observed on each farm (212 MLVA types in total) after initial analysis of the results. During this analysis, one difference in a repeat region was considered as a new MLVA type as recommended by Applied Maths (personal communication). A first explanation for this variety in MLVA types could be the MLVA method itself. Small variations within the assignment of the repeat numbers could have occurred, resulting in different MLVA types. However, during each run the same positive control was used and the same results were observed each time for this strain. So, the method had a good inter and intra repeatability (data not shown). Another possible explanation for the observed variety in MLVA types is situated in the analyzed loci. It is possible that the repeat regions of the five analyzed loci are less stable and evolve faster than the loci studied during *spa* typing and PFGE. It has been reported that repeat regions of, for example surface proteins, are hypermutable with mutation rates of 10^−2^ to 10^−5^ per generation inducing adaptation to, for example, environmental changes on a short time scale. This hypermutability results in various related isolates, which was also observed here [18,19]. However, when these loci evolve too fast this could result in a “too highly” discriminatory method, which is a possibility that should be further elucidated. In addition, more thorough investigation is needed on the studied loci and the significance of their evolution.

This variety in MLVA types resulted in the need for clustering to reduce the high number of MLVA types. Two approaches were evaluated: a single and double locus approach, where the predominant MLVA type was clustered with MLVA types with one or two differences in the repeat region, respectively. When comparing both methods, some objections came up when interpreting the results of the double locus approach. The single locus approach was highly discriminatory for MRSA ST398 isolates, but with the other approach MLVA typing lost this feature. When using the double locus approach, larger MLVA clusters were obtained, resulting in less dominant clusters within a farm. For example, on farm C, two dominant clusters were observed instead of four. With the single locus approach, we detected a shift in clusters upon transport of the pigs, which wasn’t observed in the double locus approach. So, the single locus approach was chosen and allowed us to cluster closely related MLVA types and to interpret the results better. Other research groups have described other MLVA typing schemes and more importantly, other interpretation methods (for example: using a cut-off value or various settings in the computer programs upon clustering of the results). Quite often regular gel electrophoresis was used instead of capillary electrophoresis, which makes comparison with these reports difficult [20–22]. A more recent report used the MLVA method, as described by Schouls et al., on a large collection of pig isolates originating from Dutch and German farms [23]. The researchers reported that within the dominant *spa* types of CC398 various MLVA types were observed, which made them conclude that MLVA typing could be additionally used to collect more information about these isolates. The observed MLVA variety in the present study within one *spa* type and within one farm indicates that our typing scheme is even more discriminatory than the one used by Brandt et al. [23].

Nevertheless, caution is needed upon combination of the typing results. *Spa* typing, PFGE and MLVA are methods that are used to detect relationships between the isolates. For example, when generating the MST (MLVA results) the most dominant type is considered as the basal or ancestral type. Subsequently, the remaining MLVA types are positioned according to their differences in repeat regions. When using SCC*mec* typing, the horizontal gene transfer of these cassettes is studied. On farm C, two SCC*mec* cassettes were found, which indicates that two separate transfers have occurred. However, it is unknown when these transfers have happened (before or after entering of the MRSA type on the farm). Both cassettes were found in different MLVA clusters (type IV in clusters C/E and type V in clusters B/D). This would mean that the proposed relationships between the MLVA types are incorrect and should be adapted. However, seen the limited number of isolates that underwent SCC*mec* typing, more typing is needed to investigate this.

Another consideration is the definition of livestock-associated MRSA. Within the MRSA ST398 type, a distinction is made between strains of human origin and strains of animal origin. It has been reported that the φ3 bacteriophage and human specificity genes (*chp*, *sak* and *scn*) are present in the first strains and absent in the latter [24,25]. It could be interesting to investigate the presence/absence of these genes in our isolates in the future to find out the exact origin. This lays outside the scope of the present study, as we investigated possible transmission between the sows and their offspring, between the pigs and the environment.

The main objective of the present work was to gain insights into the genetic diversity of LA-MRSA isolates originating from a farm. Remarkably, the same genotype (same *spa*, pulsotype, and MLVA cluster) was common on farms B, C, and D. Direct carry-over of LA-MRSA between these farms could be excluded (geographical distance, different veterinarians, no direct exchange of animals) [10]. The *spa* type on these farms was t011, which is a more widely distributed MRSA ST398 strain in pig farms than others. It is also possible that farm specific or region specific genotypes are present, since the genotype, found on farm A was unique to this farm. Since only four farms were sampled, additional samplings are needed to elucidate these possibilities.

On each farm, a few dominant genotypes were isolated from the animals and their environment. Possibly, these dominant types persist better within the farrow-to-finish farm. For example, on farm C, four clusters were observed and all clusters were present on both sites, but the isolation percentage of the clusters was not equal on both sites: clusters B and D were isolated from less than 10% of the isolates on site 1, whereas from 30 to 40% of the isolates on site 2. Further research on more farms is needed to investigate the possibility of persistence of few types and to determine the exact mechanism for the observed persistence.

Another interesting observation is that the sows did not carry the most prevalent MLVA types, as observed in their offspring. It might be possible that these dominant MLVA types are age-specific. However, compared to the number of piglet isolates, fewer sow isolates were obtained, so, additional sow samplings and typing are needed to confirm the presence of age-specific MLVA types.

Besides the dominant MLVA types most pigs carried another MLVA type at least once throughout their lifespan. These additional types were either related or non-related to the dominant type. It has been reported before that various MRSA types can be present in one sample [26]. Other plausible hypotheses for these results are that some MLVA types are transition types to another type; that the animals are intermittent carriers of certain MLVA types or carriers of other MLVA types besides the dominant type. Because only one isolate per animal and per sampling event was analyzed, these hypotheses cannot be confirmed from the present data. In addition, the isolation method (overnight enrichment in salt-enriched broth) used in the study may have been insufficient to detect all MRSA present on the animals since other studies use two-step enrichments and antimicrobial enriched broths [10,27,28].

A second objective was to determine potential MRSA sources for the animals present at the farm. In general, a few dominant and widespread genotypes were detected in the animals and environment of the farm. Since a farrow-to-finish farm can be considered as a closed system (few animals are imported), this would mean that the farm as it whole can act as a source for newborn piglets. Using MLVA typing helped to clarify three possible LA-MRSA sources for pigs. First, in general, none of the piglets carried an identical MLVA type to its mother sow at farrowing. Nevertheless, in most cases, the MLVA types of mother sows and their offspring were closely related and belonged to the same cluster, which could indicate that the mother sow is a possible MRSA source for her offspring. This could be explained by the fast occurrence of mutations in the repeat regions of the five genes when the mother sow strains colonize their offspring. Moreover, Crombé et al. [29] reported on the presence of maternal antibodies in piglets, which puts forward the possibility of piglets being immune to the mother sow MLVA type, but not to the closely related type. During the present study, only a selection of the sow isolates was molecularly typed due to the large number of obtained isolates. It is possible that typing more sow isolates would have given additional information. Second, the dominant MLVA clusters were also found in the environment of the piglets, which could be considered as an additional source for the animals. Moreover, transmission between animals and their environment and *vice versa* was already suggested and demonstrated by other research groups [30,31]. Third, when mingling pigs upon relocation in other units, spread of some MLVA types throughout the group occurred, which confirms that the pig(let)s themselves act as a MRSA source. This was already reported in various colonization experiments [32,33].

In conclusion, during the present study isolates of animal and environmental origin were studied using MLVA typing, PFGE, *spa* typing, and SCC*mec* typing. The latter three methods demonstrated the clonal properties of the isolates, but more variation was observed when using MLVA typing. One genotype was similar on three farms, which could indicate that at least one LA-MRSA clone is more widespread than others. Within a farm, a few dominant genotypes were present (one pulsotype, one *spa* type, one SCC*mec* type and a few MLVA clusters), which were widespread. Potential MRSA sources for piglets were the mother sows, the environment, and other piglets. In conclusion, a farrow-to-finish farm can be considered as a closed system in which a dominant MRSA clone persists once entered in the farm.

## Additional files

Additional file 1:
**Minimum Spanning Tree of the MLVA results, obtained from each farm, generated after categorical analysis using UPGMA (tolerance: 0%) in Bionumerics 6.5. (farm A: green, farm B: red, farm C: light blue and farm D: dark blue).** This file shows the MST of all isolates of all farms. Additional file 2:
**Overview of the obtained MLVA results of the isolates originating from the four farms (A-D).** The MLVA types occurring on three or two farms are shown first. Afterwards, the MLVA types are ordered from farm A to D. For each MLVA type, present at the farm, the number of isolates per origin is shown. The last column indicates the isolate percentage per farm, present in the MLVA type. The pigs of farm C were transported from site 1 (C1) to site 2 (C2). The sows of farm C were only present on site 1. This file shows all the obtained MLVA types of the different farms according to their origin. Additional file 3:
**Results of the four performed molecular typing methods on a selection of isolates, originating from the four farms (A-D).** This selection, consisting of 11, 11, 25, and 24 isolates of farms A through D, respectively, represents isolates originating from the dominant MLVA and related clusters and the dominant pulsotypes. In addition, sow and piglet combinations and various sampling events were represented. For each isolate, the farm of origin, isolate origin, MLVA numeric code, MLVA cluster or MLVA type, pulsotype number, *spa* type and SCC*mec* type is shown (d: days, h: hour). This file shows the typing results of the isolates on which all typing methods were performed. Additional file 4:
**Overview of the MLVA typing results of the different sow, pig and wall isolates, originating from farm A.** The MLVA types are shown in numbers per sampling point and per animal (the 5-digit code for each MLVA type is shown in Additional file 2). Pigs are ordered according to their mother sow. No isolates were obtained within the hour and 17 days after farrowing. MLVA types belonging to the dominant cluster A are coloured in blue (d: days after farrowing). The file shows an overview of the obtained MLVA types of all the isolates of the selected animals from farm A per sampling point. Additional file 5:
**Overview of the MLVA typing results of the different sow, pig and wall isolates, originating from farm B.** The MLVA types are shown in numbers per sampling point and per animal (the 5-digit code for each MLVA type is shown in Additional file 2). Pigs are ordered according to their mother sow. No isolates were obtained 23 days after farrowing. MLVA types belonging to the dominant clusters B, F and G are coloured in orange, yellow and brown, respectively (h: hour after farrowing; d: days after farrowing). The file shows an overview of the obtained MLVA types of all the isolates of the selected animals from farm B per sampling point. Additional file 6:
**Overview of the MLVA typing results of the different sow, pig and wall isolates, originating from farm C.** The MLVA types are shown in numbers per sampling point and per animal (the 5-digit code for each MLVA type is shown in Additional file 2). Pigs are ordered according to their mother sow. The piglets resided in the growing unit of site 1 (GU-1) and were transported on day 31 to the growing unit of site 2 (GU-2). MLVA types belonging to the dominant clusters B, C, D and E are coloured in orange, green, red and light blue, respectively (h: hour after farrowing; d: days after farrowing). The file shows an overview of the obtained MLVA types of all the isolates of the selected animals from farm C per sampling point. Additional file 7:
**Overview of the MLVA typing results of the different sow, pig and wall isolates, originating from farm D.** The MLVA types are shown in numbers per sampling point and per animal (the 5-digit code for each MLVA type is shown in Additional file 2). Pigs are ordered according to their mother sow. The piglets resided in the first finishing unit for one month and were then transported to the second finishing unit. MLVA types belonging to the dominant clusters B and D are coloured in orange and red, respectively (d: days after farrowing). The file shows an overview of the obtained MLVA types of all the isolates of the selected animals from farm D per sampling point.

## Abbreviations

MLVA: Multiple-locus variable-number tandem-repeat analysis
MLST: Multilocus sequence typing
LA-MRSA: Livestock-associated MRSA
MRSA: Methicillin-resistant *Staphylococcus aureus*
MST: Minimum Spanning Tree
PFGE: Pulsed Field Gel Electrophoresis
UPGMA: Unweighted pair cluster method using averages
