# Supplementary figures and images for: Genetic diversity of livestock-associated MRSA isolates obtained from piglets from farrowing until slaughter age on four farrow-to-finish farms
Source: Vet Res. 2014 Sep 13;45(1):89. doi: 10.1186/s13567-014-0089-4 (PMC4189174; doi:10.1186/s13567-014-0089-4)

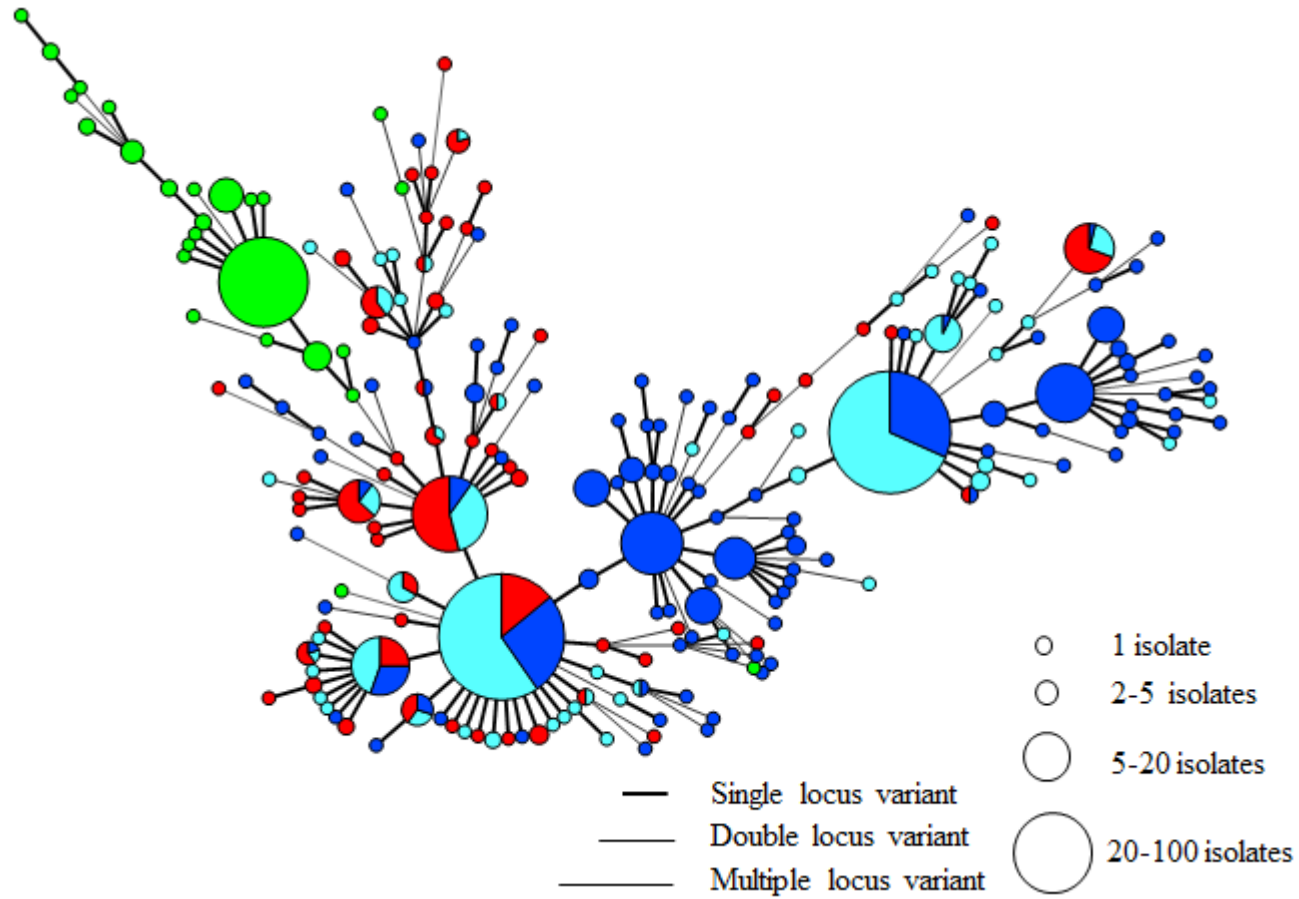

Supplement: Additional file 1: — Minimum Spanning Tree of the MLVA results, obtained from each farm, generated after categorical analysis using UPGMA (tolerance: 0%) in Bionumerics 6.5. (farm A: green, farm B: red, farm C: light blue and farm D: dark blue). This file shows the MST of all isolates of all farms. [file 13567_2014_89_MOESM1_ESM.pdf]
