# Supplementary material for: Genetic diversity of livestock-associated MRSA isolates obtained from piglets from farrowing until slaughter age on four farrow-to-finish farms
Source: Vet Res. 2014 Sep 13;45(1):89. doi: 10.1186/s13567-014-0089-4 (PMC4189174; doi:10.1186/s13567-014-0089-4)
Supplement: Additional file 2: — Overview of the obtained MLVA results of the isolates originating from the four farms (A-D). The MLVA types occurring on three or two farms are shown first. Afterwards, the MLVA types are ordered from farm A to D. For each MLVA type, present at the farm, the number of isolates per origin is shown. The last column indicates the isolate percentage per farm, present in the MLVA type. The pigs of farm C were transported from site 1 (C1) to site 2 (C2). The sows of farm C were only present on site 1. This file shows all the obtained MLVA types of the different farms according to their origin. [file 13567_2014_89_MOESM2_ESM.pdf]

MLVA numeric code[a]	MLVA Type	Number of isolates per isolate origin																		
		Sow				Pig					Wall					Isolate percentage (%)			
		A	B	C	D	A	B	C_1	C_2	D	A	B	C_1	C_2	D	A	B	C	D
33-57-36-6-3	1		0	1	2		10	0	0	0		2	0	1	3		7.7	0.6	1.7
33-57-36-7-3	2		0	0	2		9	2	7	14		0	0	2	0		5.8	3.7	5.0
33-57-37-5-3	3		0	2	1		4	0	0	0		0	1	1	2		2.6	1.1	1.0
33-57-37-6-3	4		0	4	6		29	0	0	6		4	0	2	6		21.3	1.6	5.6
33-57-37-7-3	5		0	0	0		24	15	28	99		0	0	2	2		15.5	12.7	31.3
32-55-35-5-3	6		0		2		8			1		0			1		5.2		1.2
33-57-37-4-3	7		0		3		3			0		0			3		1.9		1.9
34-59-38-8-4	8			0	0			11	40	110			0	0	0			14.4	34.1
32-46-38-38-2	9	1				83					2					63.7			
32-46-39-38-2	10	2				10					0					8.9			
32-46-38-38-3	11	0				8					0					5.9			
32-45-37-37-2	12	0				5					0					3.7			
31-45-37-37-2	13	0				2					0					1.5			
32-44-36-36-2	14	1				1					0					1.5			
32-46-37-37-2	15	0				2					0					1.5			
32-46-38-37-2	16	0				2					0					1.5			
31-53-34-1-2	17		1				3					0					2.6		
33-57-36-4-3	18		1				2					0					1.9		
33-57-37-3-3	19		0				3					0					1.9		
32-55-35-3-3	20		0				2					0					1.3		
32-55-35-4-3	21		0				2					0					1.3		
33-47-37-6-3	22		0				2					0					1.3		
33-51-36-7-3	23		0				2					0					1.3		
33-52-36-7-3	24		0				2					0					1.3		
33-57-35-6-3	25		0				1					1					1.3		
32-53-35-5-3	26		0				2					0					1.3		

MLVA numeric code[a]	MLVA Type	Number of isolates per isolate origin																	
		Sow				Pig					Wall					Isolate percentage (%)			
		A	B	C	D	A	B	C1	C2	D	A	B	C1	C2	D	A	B	C	D
34-57-34-7-3	27			3				34	1				3	0				11.5	
35-59-35-8-4	28			0				30	1				0	0				8.7	
34-57-33-7-3	29			0				17	1				0	0				5.1	
34-47-34-7-3	30			2				11	0				0	0				3.7	
34-57-34-6-3	31			10				1	0				2	0				3.7	
35-48-35-8-4	32			0				13	0				0	0				3.7	
35-59-38-8-4	33			0				3	3				0	0				1.6	
34-57-34-7-1	34			0				5	0				0	0				1.4	
33-55-33-6-3	35			0				3	0				0	0				0.8	
34-47-33-7-3	36			0				3	0				0	0				0.8	
34-57-37-7-3	37			0				3	0				0	0				0.8	
34-57-34-4-3	38			1				0	0				0	1				0.6	
34-57-34-5-3	39			1				0	0				1	0				0.6	
35-58-35-8-4	40			0				2	0				0	0				0.6	
35-59-35-8-2	41			0				2	0				0	0				0.6	
35-59-35-8-3	42			0				2	0				0	0				0.6	
34-58-38-8-4	43				0					13					0				4.1
34-59-38-8-3	44				0					3					0				1.0
33-55-37-7-3	45				1					1					0				0.7
34-57-38-8-4	46				0					2					0				0.7
34-59-38-7-4	47				0					2					0				0.7
Others[b]	48-212	0	2	21	5	14	35	40		25	2	1	11		6	11.8	24.5	20.3	10.9

[a]VNTR code of the repeat region of the 5 genes c*lf*A, c*lf*B, *sdr*C, *sdr*E and SIRU21)

[b]MLVA types containing only one isolate being on farm A (MLVA types 48-63): 32-45-36-36-2; 30-44-36-36-2; 32-45-37-37-1; 25-46-38-38-2; 30-46-38-38-2; 31-46-38-38-2; 32-49-38-38-2; 32-46-38-38-1; 32-46-39-38-3; 33-46-38-37-3; 33-46-38-38-3; 38-46-38-38-4; 32-43-33-33-2; 32-43-35-34-2; 25-40-39-38-3 and 33-44-34-34-3; on farm B (MLVA types 64-103): 33-57-37-38-3; 33-53-37-6-3: 33-52-37-6-3; 33-50-37-6-3; 33-48-37-6-3; 33-49-37-6-3; 33-55-37-6-3; 33-46-37-6-3; 33-46-37-7-3; 33-47-37-7-3; 33-41-37-7-3; 33-53-37-7-3; 33-51-37-7-3; 32-57-35-6-3; 33-47-36-6-3; 33-55-36-6-3; 32-57-36-6-3; 33-53-36-7-3; 33-52-36-2-3; 33-52-36-6-3; 31-54-35-3-3; 31-54-35-4-3; 32-55-35-1-2; 31-55-35-1-2; 32-51-34-1-2; 32-55-34-1-2; 32-55-34-2-2; 31-53-34-2-3; 31-

53-34-3-3; 34-52-38-8-4; 34-48-38-8-4; 33-53-37-5-3; 33-53-33-8-3; 33-51-30-9-3; 26-51-32-2-2; 27-50-32-0-2; 29-52-33-3-2; 31-53-33-3-2; 28-51-33-0-2 and 33-55-36-1-2; on farm C (MLVA types 104-176): 34-57-37-6-3; 34-57-37-5-3; 34-57-33-4-3; 34-57-33-6-3; 34-57-33-3-3; 34-57-33-5-3; 34-57-36-7-3; 34-57-34-8-3; 34-57-34-2-3; 34-57-34-3-3; 34-53-34-7-3; 34-58-34-7-3; 34-47-34-5-3; 34-51-34-6-3; 34-51-34-7-3; 34-57-33-7-1; 34-48-34-7-1; 33-58-37-7-3; 33-57-33-7-3; 33-57-36-4-3; 33-57-36-5-3; 32-55-35-6-3; 32-57-35-6-3; 33-55-35-1-3; 33-55-35-0-3; 33-46-33-6-3; 27-51-33-6-3; 27-49-33-6-3; 27-50-33-6-3; 34-57-34-5-2; 35-57-34-5-2; 35-58-38-8-4; 34-58-38-8-4; 34-52-38-8-4; 34-56-38-8-4; 26-59-38-8-4; 31-53-33-8-4; 31-53-35-8-4; 36-62-39-8-4; 35-59-39-8-4; 33-57-37-8-4; 33-57-36-8-4; 34-57-39-11-3; 29-52-32-7-3; 28-51-32-7-3; 28-49-34-6-3; 29-50-34-6-3; 24-46-30-6-3; 31-53-35-6-3; 33-55-33-2-3; 24-57-32-6-1; 32-53-34-4-3; 32-54-34-9-4; 34-58-34-2-2; 34-57-33-8-2; 36-59-34-1-2; 30-57-31-4-2; 29-39-30-2-2; 32-55-32-3-2; 35-59-35-9-4; 35-47-35-8-2; 35-58-35-7-4; 35-50-38-8-4; 36-50-36-8-4; 26-49-30-8-4; 34-57-34-8-4; 33-53-34-8-4; 33-57-38-38-4; 41-66-37-8-4; 40-65-35-9-4; 35-59-40-40-6 and 35-62-39-8-4; on farm D (MLVA types 177-212): 32-54-34-6-3; 32-55-34-6-3; 32-52-34-6-3; 32-55-35-7-3; 33-54-36-7-3; 33-58-36-7-3; 33-57-36-4-3; 33-57-36-3-3; 28-57-36-7-3; 33-51-37-7-3; 33-50-37-7-3; 33-57-37-1-3; 33-57-37-8-3; 32-57-37-7-3; 33-57-37-7-4; 33-57-37-8-4; 34-59-37-7-4; 34-58-38-7-4; 34-59-38-8-2; 34-58-38-8-3; 33-58-38-8-3; 29-52-33-1-2; 28-52-33-1-2; 33-55-36-6-3; 34-58-36-6-3; 32-55-35-1-2; 29-52-35-5-3; 29-52-34-7-3; 29-58-33-5-3; 33-57-35-6-3; 35-57-37-7-4; 33-58-35-8-4; 32-53-35-8-4; 31-55-34-8-4; 40-65-38-8-5 and 31-53-34-1-2.
