# Supplementary material for: Genetic diversity of livestock-associated MRSA isolates obtained from piglets from farrowing until slaughter age on four farrow-to-finish farms
Source: Vet Res. 2014 Sep 13;45(1):89. doi: 10.1186/s13567-014-0089-4 (PMC4189174; doi:10.1186/s13567-014-0089-4)
Supplement: Additional file 3: — Results of the four performed molecular typing methods on a selection of isolates, originating from the four farms (A-D). This selection, consisting of 11, 11, 25, and 24 isolates of farms A through D, respectively, represents isolates originating from the dominant MLVA and related clusters and the dominant pulsotypes. In addition, sow and piglet combinations and various sampling events were represented. For each isolate, the farm of origin, isolate origin, MLVA numeric code, MLVA cluster or MLVA type, pulsotype number, spa type and SCCmec type is shown (d: days, h: hour). This file shows the typing results of the isolates on which all typing methods were performed. [file 13567_2014_89_MOESM3_ESM.pdf]

Farm	Isolate origin	MLVA numeric code[a]	MLVA cluster/type[b]	Pulsotype number	*Spa* type	SCC*mec* type
A	pig 2, d187	32-46-38-38-2	A	II	t567	NT type 3
	pig 51, d3	32-46-39-38-2	A	II	t567	NT type 3
	pig 51, d5	32-46-39-38-2	A	II	t567	NT type 3
	pig 2, d7	32-45-36-36-2	48	II	t567	NT type 3
	sow 6, d7	32-44-36-36-2	14	II	t567	NT type 3
	pig 51, d7	30-44-36-36-2	49	II	t567	NT type 3
	pig 2, d52	32-46-38-38-2	A	II	t567	NT type 3
	pig 51, d52	32-46-38-38-2	A	II	t567	NT type 3
	pig 2, d75	32-46-38-38-2	A	II	t567	NT type 3
	pig 51, d75	32-46-38-38-2	A	II	t567	NT type 3
	sow 10, d1	32-46-39-38-2	A	III	t567	NT type 3
B	sow 4, h1	31-54-35-4-3	85	I	t011	V
	pig 40, d1	33-57-36-6-3	B	I	t011	V
	pig 83, d1	33-57-37-7-3	B	I	t011	V
	pig 40, d3	28-51-33-0-2	102	I	t011	V
	pig 83, d3	32-55-35-5-3	F	I	t011	V
	sow 4, d5	33-57-36-4-3	F	I	t011	V
	pig 40, d58	33-48-37-6-3	B	I	t011	V
	pig 83, d58	33-57-37-3-3	B	I	t011	V
	pig 18, d67	32-57-36-6-3	80	I	t011	V
	pig 40, d67	33-57-37-4-3	B	I	t011	V
	pig 83, d165	32-55-34-2-2	90	I	t011	V
C	sow 1, d3	33-57-37-6-3	B	I	t011	V
	sow 1, d17	33-57-36-6-3	1	I	t011	V
	pig 6, d1	33-57-37-7-3	B	I	t011	V
	pig 6, d3	33-57-36-7-3	B	I	t011	V
	pig 6, d5	34-59-38-8-4	D	I	t011	V
	pig 6, d7	31-53-35-6-3	152	I	t011	V
	pig 6, d17	34-59-38-8-4	C	I	t011	V
	pig 6, d33	34-59-38-8-4	C	I	t011	V

Farm	Isolate origin	MLVA numeric code[a]	MLVA cluster/type[b]	Pulsotype	*Spa* type	SCC*mec* type
C	pig 6, d54	33-57-37-7-3	B	I	t011	V
	pig 6, d88	33-57-37-7-3	B	I	t011	V
	pig 88, d33	33-57-37-7-3	B	I	t011	V
	pig 88, d54	34-59-38-8-4	D	I	t011	V
	pig 88, d88	34-59-38-8-4	D	I	t011	V
	pig 88, d172	26-59-38-8-4	139	I	t011	V
	sow 1, d1	33-55-35-1-3	127	I	t011	IV
	sow 1, d5	33-53-34-8-4	171	I	t011	IV
	pig 6, d21	34-57-34-7-3	C	VII	t011	IV
	sow 9, h1	32-53-34-4-3	155	V	t011	IV
	pig 88, h1	35-48-35-8-4	E	I	t011	IV
	pig 88, d1	34-47-34-7-3	D	IV	t011	IV
	pig 88, d3	34-47-34-7-3	D	I	t011	IV
	pig 88, d5	34-47-34-7-3	D	I	t011	IV
	pig 88, d7	34-47-33-7-3	D	I	t011	IV
	pig 88, d17	34-47-34-7-3	D	I	t011	IV
	pig 88, d21	35-59-35-8-4	163	I	t011	IV
D	pig 20, d3	33-57-37-6-3	B	I	t011	V
	sow 1, d6	32-55-35-5-3	6	I	t011	V
	sow 2, d6	33-57-37-6-3	B	I	t011	V
	pig 20, d6	33-57-37-7-3	B	I	t011	V
	sow 3, d6	33-57-37-6-3	B	I	t011	V
	pig 23, d6	33-57-36-7-3	B	I	t011	V
	pig 100, d6	33-55-37-7-3	B	I	t011	V
	sow 1, d20	33-57-36-6-3	1	I	t011	V
	sow 3, d20	33-57-36-4-3	182	I	t011	V
	pig 23, d20	34-59-38-8-4	D	I	t011	V
	pig 4, d27	33-57-37-7-3	B	I	t011	V
	pig 20, d27	33-58-35-8-4	207	I	t011	V
	pig 23, d27	33-55-36-6-3	199	I	t011	V
	pig 4, d35	33-57-37-7-3	B	I	t011	V

Farm	Isolate origin	MLVA numeric code[a]	MLVA cluster/type[b]	Pulsotype	*Spa* type	SCC*mec* type
D	pig 20, d35	34-59-38-8-4	D	I	t011	V
	pig 23, d35	34-59-38-8-4	D	I	t011	V
	pig 20, d62	33-57-37-7-3	B	I	t011	V
	pig 23, d77	33-57-37-7-3	B	I	t011	V
	pig 20, d159	33-57-37-7-3	B	I	t011	V
	pig 23, d159	33-57-37-7-3	B	I	t011	V
	pig 23, d48	34-59-38-8-4	D	VIII	t011	V
	pig 23, d62	34-59-38-8-4	D	VIII	t011	V
	pig 20, d108	32-55-35-7-3	179	VIII	t011	V
	pig 23, d108	33-57-37-7-3	B	VIII	t011	V

[a] VNTR code of the repeat region of the 5 genes *clf*A, *clf*B, *sdr*C, *sdr*E and SIRU21

[b] In case of a clustered MLVA type, the cluster letter is given. In case of a non-clustered MLVA type, the unique MLVA number is given.
