# Supplementary material for: Genetic diversity of livestock-associated MRSA isolates obtained from piglets from farrowing until slaughter age on four farrow-to-finish farms
Source: Vet Res. 2014 Sep 13;45(1):89. doi: 10.1186/s13567-014-0089-4 (PMC4189174; doi:10.1186/s13567-014-0089-4)
Supplement: Additional file 4: — Overview of the MLVA typing results of the different sow, pig and wall isolates, originating from farm A. The MLVA types are shown in numbers per sampling point and per animal (the 5-digit code for each MLVA type is shown in Additional file 2). Pigs are ordered according to their mother sow. No isolates were obtained within the hour and 17 days after farrowing. MLVA types belonging to the dominant cluster A are coloured in blue (d: days after farrowing). The file shows an overview of the obtained MLVA types of all the isolates of the selected animals from farm A per sampling point. [file 13567_2014_89_MOESM4_ESM.doc]

|  | MLVA results | | | | | | | | | |  | | |
| --- | --- | --- | --- | --- | --- | --- | --- | --- | --- | --- | --- | --- | --- |
| Isolate  origin | Nursing unit | | | |  | Growing unit | |  | Finishing unit | | |  |  |
|  | d1 | d3 | d5 | d7 |  | d28 | d52 |  | d75 | d187 | |  |  |
| sow 1 |  |  |  |  |  |  |  |  |  |  | |  |  |
| pig 2 |  |  |  | 48 |  |  | 9 |  | 9 | 9 | |  |  |
| pig 3 |  |  | 16 |  |  |  | 9 |  |  |  | |  |  |
| pig 6 |  |  |  |  |  |  | 12 |  |  | 14 | |  |  |
| pig 7 |  |  |  |  |  |  | 9 |  | 9 |  | |  |  |
| pig 8 |  |  |  |  |  |  | 9 |  | 59 |  | |  |  |
| sow 2 |  |  |  |  |  |  |  |  |  |  | |  |  |
| pig 11 |  |  |  |  |  |  | 9 |  | 11 |  | |  |  |
| pig 12 |  |  |  |  |  |  | 9 |  | 52 | 11 | |  |  |
| sow 3 |  |  |  |  |  |  |  |  |  |  | |  |  |
| pig 17 |  |  |  |  |  |  | 9 |  | 9 | 9 | |  |  |
| pig 19 |  |  |  |  |  |  | 9 |  | 9 |  | |  |  |
| pig 22 |  |  |  |  |  |  | 50 |  | 9 | 11 | |  |  |
| pig 26 |  |  |  |  |  |  | 9 |  | 9 |  | |  |  |
| sow 4 |  |  |  |  |  |  |  |  |  |  | |  |  |
| pig 28 |  |  |  |  |  |  | 9 |  | 9 | 9 | |  |  |
| pig 29 |  |  |  |  |  |  | 10 |  | 9 |  | |  |  |
| pig 30 |  |  |  |  |  |  | 9 |  | 9 |  | |  |  |
| pig 35 |  |  | 9 |  |  |  | 9 |  | 9 | 57 | |  |  |
| pig 36 |  |  | 9 |  |  |  | 10 |  | 9 | 12 | |  |  |
| pig 37 |  |  | 10 |  |  |  |  |  | 9 | 9 | |  |  |
| sow 5 |  |  |  |  |  |  |  |  |  |  | |  |  |
| pig 41 |  |  |  |  |  |  | 9 |  | 9 | 9 | |  |  |
| pig 42 |  |  |  |  |  |  | 10 |  | 9 |  | |  |  |
| pig 43 |  |  |  |  |  |  | 9 |  | 9 | 9 | |  |  |
| pig 44 |  |  |  |  |  |  | 9 |  |  | 56 | |  |  |
| pig 45 |  |  |  |  |  |  | 10 |  |  |  | |  |  |
| sow 6 |  |  |  | 14 |  |  |  |  |  |  | |  |  |
| pig 49 |  |  |  | 12 |  |  | 10 |  | 9 |  | |  |  |
| pig 51 |  | 10 | 10 | 49 |  |  | 9 |  | 9 |  | |  |  |
| sow 7 |  |  |  |  |  |  |  |  |  |  | |  |  |
| pig 57 |  |  |  | 9 |  |  | 12 |  | 9 |  | |  |  |
| pig 58 |  |  |  | 9 |  |  | 15 |  | 9 |  | |  |  |
| pig 61 |  |  |  |  |  |  | 9 |  |  | 9 | |  |  |
| sow 8 |  |  |  |  |  |  |  |  |  |  | |  |  |
| pig 67 |  |  |  |  |  |  | 9 |  |  | 9 | |  |  |
| pig 68 |  |  |  |  |  |  | 9 |  | 55 | 9 | |  |  |
| pig 73 |  |  |  |  |  |  | 10 |  | 9 | 9 | |  |  |
| sow 9 |  |  |  |  |  |  |  |  |  |  | |  |  |
| pig 74 |  |  |  |  |  |  | 9 |  | 9 | 51 | |  |  |
| pig 75 |  |  |  |  |  |  | 9 |  | 9 | 9 | |  |  |
| pig 78 |  |  |  |  |  |  | 10 |  | 9 | 9 | |  |  |
| pig 81 |  |  |  |  |  |  | 16 |  |  | 62 | |  |  |
| sow 10 | 10 |  |  |  |  |  |  |  |  |  | |  |  |
| pig 89 |  |  |  |  |  | 11 | 53 |  | 9 | 54 | |  |  |
| pig 90 |  |  |  |  |  | 9 | 9 |  | 9 | 9 | |  |  |
| sow 11 |  |  | 9 |  |  |  |  |  |  |  | |  |  |
| pig 93 |  | 9 |  |  |  |  | 9 |  | 9 | 11 | |  |  |
| pig 95 |  | 13 |  |  |  | 9 | 9 |  | 9 | 11 | |  |  |
| pig 98 |  | 13 |  |  |  |  | 58 |  | 9 | 9 | |  |  |
| pig 102 |  | 61 |  |  |  |  | 9 |  | 9 | 11 | |  |  |
| sow 12 |  |  | 10 |  |  |  |  |  |  |  | |  |  |
| pig 105 | 9 |  |  |  |  |  | 12 |  | 9 |  | |  |  |
| pig 106 |  |  |  |  |  |  | 9 |  | 9 | 11 | |  |  |
| pig 107 |  |  |  |  |  |  | 9 |  | 9 | 9 | |  |  |
| pig 108 |  | 15 | 9 |  |  |  |  |  |  |  | |  |  |
| wall |  |  | 63 |  |  |  | 9 |  | 9 | 60 | |  |  |
