# Supplementary material for: Genetic diversity of livestock-associated MRSA isolates obtained from piglets from farrowing until slaughter age on four farrow-to-finish farms
Source: Vet Res. 2014 Sep 13;45(1):89. doi: 10.1186/s13567-014-0089-4 (PMC4189174; doi:10.1186/s13567-014-0089-4)
Supplement: Additional file 5: — Overview of the MLVA typing results of the different sow, pig and wall isolates, originating from farm B. The MLVA types are shown in numbers per sampling point and per animal (the 5-digit code for each MLVA type is shown in Additional file 2). Pigs are ordered according to their mother sow. No isolates were obtained 23 days after farrowing. MLVA types belonging to the dominant clusters B, F and G are coloured in orange, yellow and brown, respectively (h: hour after farrowing; d: days after farrowing). The file shows an overview of the obtained MLVA types of all the isolates of the selected animals from farm B per sampling point. [file 13567_2014_89_MOESM5_ESM.pdf]

Isolate origin	MLVA results								
	Nursing unit					Growing unit		Finishing unit	
	h1	d1	d3	d5	d7	d30	d58	d67	d165
sow 1									
pig 3		5					66		6
pig 5							5	5	
pig 6							76	5	26
pig 9		19					5	100	4
sow 2									
pig 11								95	4
pig 15							5		4
pig 18		89	64				73	80	
sow 3									
pig 21							4	5	5
pig 24							24	17	
pig 25			5				4		2
pig 30							103	1	26
sow 4	85		18						
pig 35	4	71					1		93
pig 39		5				4	67	5	
pig 40		1	102				68	7	
sow 5									
pig 43							5		5
pig 44						4		5	4
pig 45							4	5	2
pig 50									96
pig 52						19		92	
pig 54						75	4	78	3
sow 6									
pig 60							4	79	6
pig 61								1	4
pig 62							69	7	1
pig 63							22	1	4
sow 7									
pig 70						24	98		4
pig 73						7	6	4	20
pig 75						91	5		
sow 8									
pig 77			4					99	
pig 80		87					4	77	94
pig 83		5	6				19	3	90
pig 85						88	83	18	4
sow 9									
pig 88	17						21	4	
pig 89		4					70	4	4
pig 90						2	23	23	5
sow 10									
pig 97	1	3		3			1	101	4
pig 99				2				6	5
pig 103		2	65					2	
pig 105			82	5			5	17	
sow 11									
pig 108						4		86	4
pig 110						6	4	5	20
pig 115							5		2
sow 12		17		74					
pig 116		2					81	4	25
pig 119		84							18
pig 120		72	21				22	4	1
pig 121		5					2	1	
wall		25	4	4	97	4	1	1	4
