# Supplementary material for: Genetic diversity of livestock-associated MRSA isolates obtained from piglets from farrowing until slaughter age on four farrow-to-finish farms
Source: Vet Res. 2014 Sep 13;45(1):89. doi: 10.1186/s13567-014-0089-4 (PMC4189174; doi:10.1186/s13567-014-0089-4)
Supplement: Additional file 6: — Overview of the MLVA typing results of the different sow, pig and wall isolates, originating from farm C. The MLVA types are shown in numbers per sampling point and per animal (the 5-digit code for each MLVA type is shown in Additional file 2). Pigs are ordered according to their mother sow. The piglets resided in the growing unit of site 1 (GU-1) and were transported on day 31 to the growing unit of site 2 (GU-2). MLVA types belonging to the dominant clusters B, C, D and E are coloured in orange, green, red and light blue, respectively (h: hour after farrowing; d: days after farrowing). The file shows an overview of the obtained MLVA types of all the isolates of the selected animals from farm C per sampling point. [file 13567_2014_89_MOESM6_ESM.pdf]

Isolate origin	MLVA results										
	Site 1							Site 2			
	Nursing unit						GU-1	GU-2		Finishing unit	
	<1h	d1	d3	d5	d7	d17	d21	d33	d54	d88	d172
sow 1		127	4	170		1					
pig 6	5	5	2	8	152	8	27	8	5	5	
pig 8	27	27	8	8	28		27	5	5	5	
pig 10	125	5	135	36	8		30	5	8	5	
sow 2		4		104							
sow 3	27	27	31	133	31	31					
pig 22	29	27	27	28	29	27	1	5	5	174	
pig 23	27	28	28	34	27	27	28	137	5	5	
pig 28	111	165	28	120	40	28	119	142	5	126	
pig 29	27	28	27	28	28		28	28	8	8	
sow 4	31		154		31						
sow 5		31		166		31					
sow 6	4	27		114	31	31					
pig 54	27		28	29	27	27	32		8	5	163
pig 55		28	28	27	8		27	8		138	136
pig 57	31	29	42	29	29		34	5	5	2	141
pig 58	37	30		29	151		27	29	8	148	
sow 7	140	117		159	146	4					
pig 63		35	29	27	29	27	156	8	8	8	2
pig 64		42	28	27	28	8	28	8	5	168	2
pig 65		169	5		29	28	27	149	8		2
sow 8		15									
sow 9	155										
pig 86	32	129	32	33	30	33	5	27	8	143	8
pig 88	32	30	30	30	36	30	28	5	8	8	139
pig 89	30	30	32	30	167		32	8	2	8	5
sow 10	161	30	27	38	3	128					
pig 91	41	35	34	8	28	5	27	8	131		8
pig 94	28	5	28	28	130	172	5	8	8	8	
pig 99	34	27	5	41	27	5	29	33	8	8	8
pig 100	34	5	27	158	27	5	115	144	5	2	8
sow 11			107			116					
pig 102	27		32		33	36	28	8	8	5	5
pig 108		35	5	37	32	122	8		5	8	145
pig 109	27		32	32	32	5	5	8	8	5	8
pig 110		8	37	32	110	8	27	33	150	8	5
sow 12		153	30	112	109	39					
pig 111	28	40	29	29	28	28	28	33	132	5	8
pig 114		28	29	29	171	27	147	8	5	8	8
pig 115		28	27	29	173	29	28	175	8	5	2
pig 117	28	27	30	28	28	28	32		8	121	5
wall 1	118	31	27	108	160	157	164	4	38	3	124
wall 2		39	27	134		31	105	1	123	2	5
wall 3		162		113	27	3		2	4		
wall 4									5		
