# Supplementary material for: Genetic diversity of livestock-associated MRSA isolates obtained from piglets from farrowing until slaughter age on four farrow-to-finish farms
Source: Vet Res. 2014 Sep 13;45(1):89. doi: 10.1186/s13567-014-0089-4 (PMC4189174; doi:10.1186/s13567-014-0089-4)
Supplement: Additional file 7: — Overview of the MLVA typing results of the different sow, pig and wall isolates, originating from farm D. The MLVA types are shown in numbers per sampling point and per animal (the 5-digit code for each MLVA type is shown in Additional file 2). Pigs are ordered according to their mother sow. The piglets resided in the first finishing unit for one month and were then transported to the second finishing unit. MLVA types belonging to the dominant clusters B and D are coloured in orange and red, respectively (d: days after farrowing). The file shows an overview of the obtained MLVA types of all the isolates of the selected animals from farm D per sampling point. [file 13567_2014_89_MOESM7_ESM.pdf]

Isolate origin	MLVA results: Nursing unit d3	d6	d20	Growing unit d27	d35	d48	d62	Finishing unit-1 d77	d102	Finishing unit-2 d108	D159
sow 1	7	6	1								
pig 1		8	8	2	8	8	5			5	8
pig 4	8	8	5	5	5	5	5	5	43	8	8
pig 7	8	2	5	5		8	2		8		
sow 2		4									
pig 16	5	4	5	2	5	5	8	8	8	197	
pig 17	8	8	181	8	43	5	8			8	5
pig 20	4	5	177	208	8	5	5			180	5
sow 3	188	4	183								
pig 23	178	2	8	200	8	8	8	5		5	5
pig 24	8	8	8	201	5	5	5	182		8	8
pig 26	196	8	8	2	5	5	43	8		8	5
sow 4		4									
pig 32	8	5	5	5	8	5	43	43	5	195	8
pig 33	8	5		5	2	5	8			5	5
pig 37	4	203	4	5	5	8	7	207			8
pig 39	5		43	5	43	8	8				211
sow 5		7									
sow 6	45	1	2								
pig 52	5	4	44		8	8	8	204			
pig 53	5	5	189	5	8	5	8	5	5	8	5
pig 56	191	5	8	8	5	8	5	5	8		5
pig 57	5	43	5	2	8	8	5				
sow 7	4	2	212								
pig 64	8	4	8	8		8	5	5	2	5	2
pig 65	8	46	8	8	5	2	5	8	192	8	5
pig 70		5	8	8	5	5	8	8	8	5	8
sow 8		198									
sow 9	4	7									
pig 82	8	8	5	5	8		8	8	5		5
pig 84	2	47	206	5			5	5	5	8	8
pig 86	5	5	8		8	8	8	5	43	8	
pig 87	190	194	8	8	5	5	8		43		8
sow 10		4									
pig 93	8	43		8	8	8	5	210	8	5	8
pig 99	44	8		8	8	2	8		5		8
pig 100	5	45		5	8	5	8	5	209		6
sow 11		6									
sow 12	205	3									
pig 114	5	44		179	8	8	5	193	5		8
pig 115	8	5		8	2	46	8	8	5		5
pig 116	8	43			8	8	43	5	5	8	47
wall 1	4	4	1	5	184	1	3	6	1	185	7
wall 2	7	7	5	199	4	187	3	202	4	4	4
